# Modified systemic inflammatory response syndrome and provider gestalt predicting adverse outcomes in children under 5 years presenting to an urban emergency department of a tertiary hospital in Tanzania

**DOI:** 10.1186/s41182-019-0136-y

**Published:** 2019-02-01

**Authors:** Meera R. Nariadhara, Hendry R. Sawe, Michael S. Runyon, Victor Mwafongo, Brittany L. Murray

**Affiliations:** 1 Emergency Medicine Department, Muhimbili University of Health and Allied Sciences, P.O. Box 65001, Dar Es Salaam, Tanzania; 2 Emergency Medicine Department, Muhimbili National Hospital, Dar Es Salaam, Tanzania; 3 Deparment of Emergency Medicine, Carolinas Medical Centre, Charlotte, NC USA; 4 Department of Emergency Medicine, Division of Pediatric Emergency Medicine, Emory University School of Medicine, Atlanta, GA USA

**Keywords:** mSIRS, Sepsis, Pediatrics, Africa, Emergency medicine, Tanzania

## Abstract

**Background:**

Modified systemic inflammatory response syndrome (mSIRS) criteria for the pediatric population together with the provider gestalt have the potential to predict clinical outcomes. However, this has not been studied in low-income countries. We investigated the ability of mSIRS and provider gestalt to predict mortality and morbidity among children presenting to the ED of a tertiary level hospital in Tanzania.

**Methods:**

This prospective observational study enrolled a convenience sample of children under 5 years old, presenting to the Emergency Medicine Department of Muhimbili National Hospital from September 2015 to April 2016. Trained researchers used a structured case report form to record patient demographics, clinical presentation, initial provider gestalt of severity of illness, and the mSIRS criteria. Primary outcomes were 24-h mortality and overall in-hospital mortality. Data was analyzed using simple descriptive statistics, Kruskal-Wallis, Mann-Whitney *U*, and chi-squared tests.

**Results:**

We enrolled 1350 patients, median age 17 months (interquartile range 8–32 months), and 58% were male. Provider gestalt estimates of illness severity were recorded for all patients and 1030 (76.3%) had complete data for mSIRS categorization. Provider gestalt classified 97 (7.2%) patients as healthy, 546 (40.4%) as mildly ill, 457 (33.9%) as moderately ill, and 250 (18.5%) as severely ill. Of the patients, classifiable by mSIRS, 411/1030 (39.9%) had ≥ 2 mSIRS criteria. In predicting 24-h mortality, the ≥ 2 mSIRS and gestalt “severely ill” had sensitivities of 82% and 81%, respectively, and specificity of 61% and 84%, respectively. In predicting overall in-hospital mortality, the ≥ 2 mSIRS and gestalt “severely ill” had sensitivities of 66% and 70% with a specificity of 62% and 86% respectively.

**Conclusion:**

Both the mSIRS and provider gestalt were highly specific for predicting 24-h and overall in-hospital mortality in our patient population. The clinical utility of these assessment methods is limited by the low positive predictive value.

**Electronic supplementary material:**

The online version of this article (10.1186/s41182-019-0136-y) contains supplementary material, which is available to authorized users.

## Background

Rapid early evaluation and treatment of critically ill children has the potential to decrease mortality in resource-limited healthcare facilities [1–4]. Although Tanzania has reduced under-five mortality from 165 to 49 deaths per 1000 live births from 1990 to 2015, the burden still exists [5, 6]. Currently, top causes of under-five mortality in Tanzania are from acute illnesses like pneumonia, malaria, and diarrhoeal diseases, conditions that are responsive to rapid identification and appropriate timely interventions [7, 8]. The modified systemic inflammatory response syndrome (mSIRS) criteria and provider gestalt are recognized assessment tools providing critical information to guide early, lifesaving interventions [9–15].

The mSIRS criteria and provider gestalt are widely used to predict the severity of illnesses in patients across the globe [16]. Where some countries utilize the predictive value of the mSIRS criteria for severity of illness and outcome in patients, some utilize provider gestalt to a greater degree, yet others use them in combination to predict the severity of illness and make management decisions in pediatric patients. Multiple studies documented in the past have established the significance of the mSIRS criteria and/or provider gestalt in better predicting the severity of illness in patients seen at emergency departments (EDs) [17].

In high-income countries, several studies have been published on the role of mSIRS criteria and provider gestalt to predict various outcomes but none so specifically documents which defined clinical outcome has been elaborately associated with a defined vital signs and/or clinicians’ gestalt and to what degree of severity of illness. The gap still exists to demonstrate and explore further on the accuracy of the mSIRS criteria and providers’ gestalt on the severity of illness and pediatric patient outcome. However, there is a lack of data on the accuracy and utility of mSIRS and provider gestalt in low-income countries to risk stratify these pediatric patients.

In Tanzania, the opening of the Emergency Medicine Department (EMD) at Muhimbili National Hospital (MNH) provided an opportunity for an improved care of critically ill children; however, as in most of developing countries, the role of mSIRS criteria and providers’ gestalt in the assessment of severity of illness in pediatric patients under the age of five presenting to the department had never been studied. We aimed to describe the predictive value of the mSIRS criteria and providers’ gestalt with regards to the severity of illness in children under the age of 5 years presenting to an urban emergency department of a tertiary hospital in Tanzania.

## Methods

### Study design

We conducted a prospective descriptive study of pediatric patients, 28 days to 5 years of age presenting to an urban tertiary EMD at MNH in Tanzania between September 2015 and April 2016. Children less than 28 days old were not included as they are seen at the pediatric neonatal ward and are not cared for in our EMD.

### Study setting and population

MNH is a 1500-bed tertiary academic hospital. The EMD acts as an entry point for most patients admitted. Clinical care at EMD is under the supervision of locally trained emergency physician who staff the EMD 24 h every day and oversee care provided by intern doctors (new graduates from medical school training), medical officers (completed 1 year of internship), and emergency medicine residents (formally masters of medicine training after 1–2 years of practice as medical officer). The nursing care is under the supervision of critical care trained nurses, who oversee nursing care provided by degree and diploma trained nurses. Approximately 200 patients a day are seen at the EMD out of which 25% are pediatric patients. There is no pediatric intensive care unit (ICU) at the MNH. Pediatric patients who are critically ill may be admitted to the Acute Pediatric Care Unit (APCU) which only has six beds. EMD policy is to have a pediatric consult for such critically ill children to be able to be admitted to the APCU, and in the event that the APCU is fully occupied, these critically ill children are sent to the general wards for care. Therefore, in this study, we considered the provider obtaining a pediatric consult as a surrogate to the provider believing the child required a higher level of care.

Patients under the age of 5 years were enrolled consecutively during specified data collection times that were purposefully selected to represent all times of the night and day as well as weekends and weekdays. All patients under the age of 5 years presenting during data collection times were screened for inclusion in the study. Written informed consent was requested from patients’ parents/guardians. Patients whose parents/guardians denied consent, those who were deemed dead on arrival, and those who had trauma were excluded.

Upon enrolment to the study, a structured data collection tool was used to collect data. Basic demographic information, vital signs and mental status upon arrival, provider gestalt at the time of initial patient evaluation, laboratory results, and patient disposition status from the EMD (discharged home, admitted to the ward/APCU, or died) were documented. We used modified systemic inflammatory response syndrome (mSIRS) criteria as two of these five clinical parameters or all: heart rate more than age-specific beats per minute, respiratory rate more than age-specific breaths per minute, a temperature > 38.5 °C or < 36 °C, total white cell count more than age-specific per cubic millimeter or > 10% immature neutrophils, and systolic blood pressure less than age-specific in millimeters of mercury [18].

Patients were then followed up at 24 h and daily until discharge, death in the hospital, or reaching a length of stay (LOS) of 30 days.

### Key outcome measures

The primary outcomes were 24-h and overall in-hospital mortality. Secondary outcomes were the need for a higher level of care as represented by admission to the APCU/need for pediatric consult in the ED, EMD mortality, and hospital LOS.

### Data analysis

Data were summarized with descriptive statistics, including the counts and percentages and medians and interquartile ranges (IQR). Ninety-five percent confidence intervals (CI) are presented where appropriate. The chi-square test or Fisher’s exact test were used to compare categorical variables, while the Mann-Whitney *U* test was used to compare continuous variables. Outcomes were analyzed comparing the ≥ 2 mSIRS and < 2 mSIRS categories and comparing between the healthy, mild, and moderate and severe gestalt categories. Loss to follow-up at 24 h were considered as lost to follow-up as there was no way to know if the patient was not registered in the ward, died, or discharged. However, patients lost to follow-up between 24 h and 30 days were considered alive because we could not find them in the mortality data records.

The diagnostic test characteristics and area under the receiver operating characteristic (ROC) curve were calculated for the mSIRS and provider gestalt categories using Microsoft Excel 2016 (Microsoft Corporation, Redmond, WA, USA) and StatsDirect (version 3.0.167, StatsDirect Ltd., Cheshire, UK).

## Results

**Fig. 1:**
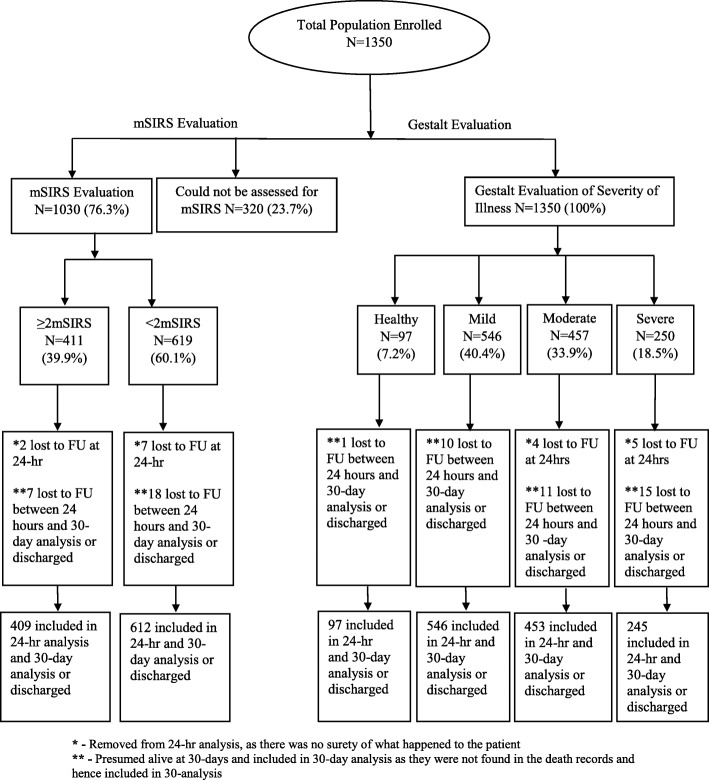
Flow diagram

We enrolled 1350 pediatric patients, 320 (23.70%) could not be categorized as per the mSIRS criteria due to the lack of white blood cell counts. Out of the total 1030 (76.30%) children that had available data to determine the mSIRS criteria, 411 (39.90%) had ≥ 2 mSIRS criteria and 619 (60.10%) had < 2 mSIRS. All patients were evaluated by provider gestalt, with 97 (7.19%) patients categorized as healthy, 546 (40.44%) as mildly ill, 457 (33.85%) as moderately ill, and 250 (18.52%) as severely ill. Overall, 9 (0.67%) patients were lost to follow-up at 24 h and 37 (2.74%) were lost to follow-up at 30-day analysis or upon being discharged from hospital (Fig. 1).

### Baseline demographic characteristics of the study population

The median age of our population was 17 months (IQR 8–32 months) with a male predominance (784/1350, 58.07%). Children categorized into being assessable by the mSIRS criteria were found to be younger than those who could not be categorized by the mSIRS criteria (*p* < 0.0001) and were more likely to present with hypoxia (Table 1).

**Table 1:** Baseline demographic characteristics of the study population

	All (*N* = 1350)	Classifiable by mSIRS (*n* = 1030)	Not classifiable by mSIRS (*n* = 320)	*p* value
Age, months				
Median (interquartile range)	17 (8–32)	15 (7–28)	24 (9–41)	< 0.0001
Gender				
Male, no. (%)	784 (58.07%)	599 (58.16%)	185 (57.81%)	0.91**
Female, no. (%)	566 (41.93%)	431 (41.84%)	135 (42.19%)	
Findings at presentation				
SBP (mmHg)				
Median	102	101	102	0.95*
Interquartile range	92–111	93–110	91–111	
Hypotension, no. (%)^†^	67 (4.96%)	54 (5.24%)	13 (4.06%)	0.40**
Pulse rate				
Median	138	142	127	< 0.0001*
Interquartile range	120–156	123–158	113–143	
Tachycardia, no. (%)^‡^	231 (17.11%)	212 (20.58%)	19 (5.94%)	< 0.0001**
Bradycardia, no. (%)^∫^	26 (1.93%)	20 (1.94%)	6 (1.88%)	0.94**
Respiratory rate				
Median	32	32	32	0.48*
Interquartile range	26–35	26–34	28–36	
Abnormal temperature, no. (%)^ǁ^	232 (17.19%)	139 (13.50%)	32 (10%)	0.10**
Oxygen saturation < 95%, no. (%)	98 (7.26%)	89 (8.64%)	9 (2.81%)	0.0004**
Level of responsiveness				< 0.0001**
Alert, no. (%)*	1274 (94.37%)	956 (92.82%)	318 (99.38%)	
Voice, no. (%)**	12 (0.89%)	12 (1.16%)	0	
Pain, no. (%)^⁑^	47 (3.48%)	46 (4.47%)	1 (0.31%)	
Unresponsive, no. (%)§	17 (1.26%)	16 (1.55%)	1 (0.31%)	

^a^Hypotension was defined as systolic blood pressure of less than 75 mmHg in children aged 1 month to more than 5 years and less than 74 mmHg in children aged 2 to 5 years

^‡^Tachycardia was a heart rate of more than 180 beats per minute in children aged 1 month to less than 2 years and more than 140 bpm in children aged 2 to 5 years

^∫^Bradycardia was defined as less than 90 bpm in children aged 1 month to less than 2 years, and it was not applicable to children aged 2 to 5 years

^ǁ^Temperate was defined as measuring at the axilla, either less than 35.4 °C or more than 37.9 °C

*Alertness in a child was defined as the child being active

**Responding to voice was defined as the child responded when called his/her name by the parent/guardian in children aged less than 1 year and child responding by making sound or words in children aged more than 1 year

^⁑^Painful stimulus in a child was defined as when the child was touched or inflicted pain, cried, or grimaced

^§^Unresponsiveness was defined as the child not responding to any external stimulus

Children classified into the severely ill category were more likely to be younger, have altered mental status, and be febrile when compared to all other gestalt categories (all *p* < 0.05). Patients in the severe gestalt category were also more likely to be hypotensive than those in the healthy category (*p* = 0.0462) (Additional file 1: Table S1).

### Primary and secondary outcomes

Patients with the ≥ 2 mSIRS criteria were more likely to have adverse events than those with < 2 mSIRS criteria: 24-h mortality in the ≥ 2 mSIRS group was 27/409 (6.60%, 95% CI 4.19 to 9.01%) versus 6/612 (0.98%, 95% CI 0.20 to 1.80%) in the < 2 mSIRS group (*p* < 0.0001), and overall in-hospital mortality analyzed at 30 days in the ≥ 2 mSIRS group was 65/409 (15.90%, 95% CI 12.30 to 19.40%) versus 33/612 (5.39%, 95% CI 3.60 to 7.20%) in the < 2 mSIRS group (*p* = < 0.0001). ED mortality in the ≥ 2 mSIRS group was 9/411 (2.19%, 95% CI 0.78 to 3.60) versus in the < 2 mSIRS group 1/619 (0.16%, 95% CI − 0.15 to 0.47%) (*p* = 0.0012). There were no significant differences between children with ≥ 2 mSIRS and < 2 mSIRS in relation to need for pediatric consultation in the ED/APCU admission or hospital LOS (Table 2).

**Table 2:** Primary and secondary outcomes in the ≥ 2 mSIRS and < 2 mSIRS categories

Category	≥ 2 mSIRS			< 2 mSIRS			*p* value
Primary outcomes	*N* = 411			*N* = 619			
	*n*	%	95% CI	*n*	%	95% CI	
24-h mortality	27	6.6	4.2–9.0	6	0.98	0.2–1.8	< 0.0001
Mortality (in hospital)	65	15.9	12.5–19.8	33	5.4	3.6–7.2	< 0.0001
Secondary outcomes		*N* = 411			*N* = 619		
APCU admission/pediatric consult	22	5.4	3.2–7.6	20	3.2	1.8–4.6	0.09
ED mortality	9	2.2	0.8–3.6	1	0.2	0.15–0.55	0.001
LOS	4 (IQR 1–8)			2 (IQR 0–7)			

### Primary and secondary outcomes by provider gestalt category

All 12 children who died in the ED were categorized into the severe provider gestalt category. All patients categorized as healthy survived throughout the study period. At 24-h follow-up, 2/546 (0.37%, 95% CI − 0.14 to 0.88%) patients categorized as mildly ill died, compared with 5/453 (1.10%, 95% CI 0.14 to 2.06%) patients in the moderately ill category and 30/245 (12.24%, 95% CI 8.14 to 16.34%) patients in the severely ill category (*p* = < 0.0001).

Overall in-hospital mortality was 10/546 (1.83%, 95% CI 0.70 to 3.0%) in the mildly ill category, 22/453 (4.86%, 95% CI 2.90 to 6.80%) in the moderately ill category, and 75/245 (30.61%, 95% CI 24.80 to 36.40%) in the severely ill category (*p* < 0.0001).

The need for pediatric consult in the ED or APCU admission was significantly higher in the severe gestalt category versus all other gestalt categories (all *p* < 0.0001). LOS was significantly different between all provider gestalt categories and it increased with increasing severity of gestalt categorization (*p* < 0.0001) (Table 3).

**Table 3:** Primary and secondary outcomes by provider gestalt category

Outcome	Healthy	Mild	Moderate	Severe	*p* value
Primary outcomes					
	*N* = 97	*N* = 546	*N* = 453	*N* = 245	
24-h mortality	0	2 (0.4%, 95% CI − 0.13 to 0.93%)	5 (1.1%, 95% CI 0.14–2.06%)	30 (12.2%, 95% CI 8.10–16.30%)	< 0.0001
In-hospital mortality	0	10 (1.8%, 95% CI 0.70–3.00%)	22 (4.9%, 95% CI 2.90–6.80%)	*75* (*30.6*%, *95*% *CI 24.80*–*36.40*%)	< 0.0001
Secondary outcomes					
	*N* = 97	N = 546	*N* = 457	*N* = 250	
APCU admission/pediatric consult	2 (2.1%, 95% CI −0.75 to 4.95%)	6 (1.1%, 95% CI 0.23–1.97%)	14 (3.1%, 95% CI 1.51–4.69%)	24 (9.6%, 95% CI 5.95–13.25%)	< 0.0001
ED mortality	0	0	0	12 (4.8%, 95% CI 2.15–7.45%)	< 0.0001
LOS	0 IQR 0–0	0 IQR 0–4	3 IQR 0–7.8	6.5 IQR 2–13	< 0.0001

*Removed from the 24-h analysis, as there was no surety of what happened to the patient

**Presumed alive at 30 days and included in the 30-day analysis as they were not found in the death records and hence included in 30-analysis

For 24-h mortality, the ≥ 2 mSIRS criteria had a sensitivity of 82%, a specificity of 61%, a PPV of 6.60%, a positive likelihood ratio (LR+) of 2.12, and a negative likelihood ratio (LR−) of 0.30. For overall in-hospital mortality, the ≥ 2 mSIRS had a sensitivity of 66%, a specificity of 62%, a PPV of 16%, LR+ of 1.78, and LR− of 0.54.

**Fig. 2:**
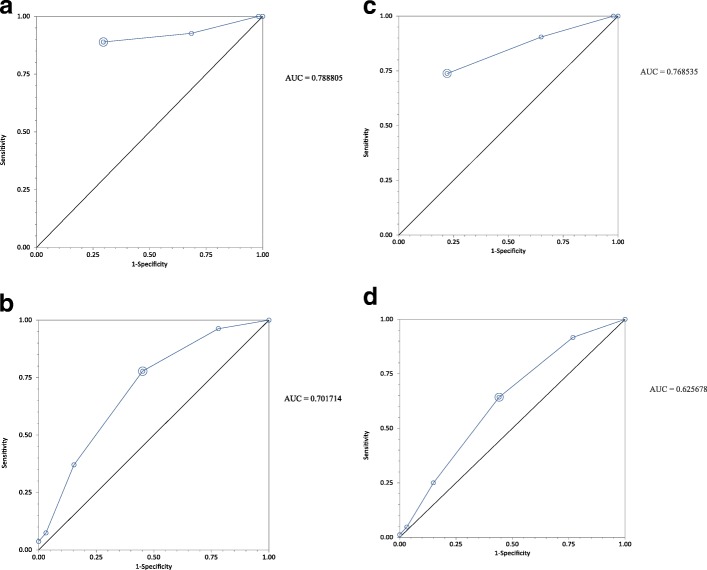
**a** The ROC curve for provider gestalt to predict 24-h mortality. **b** The ROC curve for mSIRS criteria to predict 24-h mortality. **c** The ROC curve for provider gestalt to predict overall in-hospital mortality. **d** The ROC curve for mSIRS criteria to predict overall in-hospital mortality

For 24-h mortality, the severe gestalt category had a sensitivity of 81%, a specificity of 84%, a PPV of 12%, a LR+ of 4.90, and a LR− of 0.23 when compared to all other categories combined. For overall in-hospital mortality, the severe gestalt category had a sensitivity of 70%, a specificity of 86%, a PPV of 30%, a LR+ of 5.00, and a LR− of 0.35 when compared to all other categories combined. The area under the receiver operating characteristic (ROC) curve for 24-h mortality in the ≥ 2 mSIRS was 0.70 (95% CI 0.61 to 0.79), and for severe provider, gestalt was 0.79 (95% CI 0.71 to 0.86). The area under the ROC curve for overall in-hospital mortality in the ≥ 2 mSIRS was 0.63 (95% CI 0.57 to 0.68), and for severe provider, gestalt was 0.77 (95% CI 0.72 to 0.82) (Fig. 2 a–d).

## Discussion

The high level of acuity seen in our population of under-fives was consistent with that found in other acute care and in-hospital settings in sub-Saharan Africa [5, 19–24]. Various other studies in sub-Saharan Africa demonstrated a large burden of critically ill children of under-fives with varying mortality and case fatality rates that are similar to the overall mortality of 7.78% found in our study [5, 22, 25, 26]. These under-five acuity levels, mortality, and admission rates are much higher than those reported from hospitals in high-resource settings [5, 6]. This combination of acuity in the under-five population and lack of resources dedicated to acute care in sub-Saharan Africa exemplifies why a mechanism to stratify children that are acutely ill into categories that may help providers triage and prioritize them more reliably is necessary [24, 27, 28]. In this study, we attempted to do this using the mSIRS and provider gestalt.

The mSIRS criteria are used to identify critically ill children that need acute intervention in the EDs and in-hospital settings globally [9, 12, 29, 30]. Although mSIRS uses criteria that are easily available during initial patient encounter, in our setting, almost 25% of the study population were unable to be assessed by the mSIRS criteria due to lack of WBC. Furthermore, a third (*n* = 411/1350) of our study population was categorized to have ≥ 2 mSIRS criteria and that means these ill children should be immediately attended to with a higher level of care. The limited resources make it difficult to cater for this large percentage of population identified as possibly critically ill in a timely manner. In countries with more resources and lower burden of critically ill children, the mSIRS criteria have been successfully used to identify children who require immediate care; however, this is possible only because there are more resources available and a lower percentage of patients classified to have the ≥ 2 mSIRS criteria [14, 31–33].

On the contrary, in line with our findings, research conducted in other areas of sub-Saharan Africa on the utilization of the mSIRS criteria has identified a high percentage of children with ≥ 2 mSIRS with a low sensitivity for having adverse outcomes which poses a challenge for health care providers to appropriately prioritize patients for timely treatment [30, 34–36]. Therefore, there is a need for criteria that can be utilized in low resource settings, which providers can use to appropriately triage and care for these patients.

Subjective methods of identifying critically ill children have also shown to be an effective way to prioritize and treat in high resource settings. Provider gestalt is one such subjective method that has shown utility in high-income countries [13, 15]. However, the sensitivity of provider gestalt has been variable, from being superior to established clinical decision rules to being inadequate or equipotent in predicting adverse outcomes [13, 15, 37–42]. In our study, the provider gestalt better predicted the adverse outcomes when compared to the mSIRS criteria; however, individually, neither had a high enough positive predictive value to be of clinical utility. Although provider gestalt predicted the adverse events better than did the ≥ 2 mSIRS criteria, they both classified too many children in the critically ill category.

Considering the above findings, we feel ≥ 2 mSIRS criteria and provider gestalt do not have high enough positive predictive value to allow the classification and rapid treatment of critically ill children in resource-limited settings because they simply identify too many children as fitting the most severe categories. We found that both mSIRS and gestalt miss an average of 1 out of 5 children who die within 24 h, which is relatively high given the severity of the outcome observed. We suggest that research to determine more objective ways to stratify these large critically ill child populations presenting to resource-limited settings that could provide timely and appropriate interventions to them. Furthermore, we considered patients identified by provider gestalt as having severe illness, who did not die, to be false positives; it is possible that some who might have died had clinicians not intervened after identifying them as being high risk.

In a limited resource setting, a tool with a slightly lower specificity and a higher sensitivity may be appropriate as it may allow more critically ill children to be treated in a timely manner with a trade-off not identifying some critically ill children. This tool would need to be carefully designed to do the best for most critically ill children and maximize the use of the available resources in these settings. The realization of this would improve care and reduce morbidity and mortality in resource-limited settings in pediatric population.

### Limitations

This study was limited by the fact that we utilized convenience sampling. We purposefully included research shifts on holidays, weekends, and all times of the day and night to represent actual population as best as possible.

Another limitation was that we chose to utilize the mSIRS criteria to categorize patients without further specific evaluation for infection that would have classified them as possible sepsis. We chose this as children without infection may also have inflammation resulting in the ≥ 2 mSIRS criteria, but it did make it difficult to compare our population to the existing literature that focuses on sepsis. It is also possible that the providers may have instinctively incorporated the knowledge of the mSIRS criteria into respective gestalt classification, so the researchers felt this classification may not have been independent of each other. Finally, this was a single-center study performed at a national hospital; this may limit the generalizability of the study results at other levels in the health care system. However, it is our hope that MNH, being a tertiary level referral hospital, that receives patients from the entire country, will be reflective of the highest level of care in the nation.

## Conclusion

The mSIRS criteria and provider gestalt both predict adverse outcomes in children presenting with acute critical illness at EMD, MNH. Provider gestalt has a better positive predictive value in predicting adverse outcomes; however, neither the mSIRS criteria nor provider gestalt had a high enough positive predictive value to be useful in resource-limited settings. There remains a clear need for better pediatric illness categorization tools purposefully designed for resource-limited settings.

## Additional files

Additional file 1: **Table S1.** Baseline Demographics of children evaluating Gestalt. (DOCX 18 kb)

## Abbreviations

APCU: Acute Pediatric Care Unit
ED: Emergency department
EMD: Emergency Medicine Department
IQR: Interquartile range
LOS: Length of stay
MNH: Muhimbili National Hospital
mSIRS: Modified systemic inflammatory response syndrome
MUHAS: Muhimbili University of Health and Allied Sciences
SIRS: Systemic inflammatory response syndrome
WHO: World Health Organization
