# Supplementary material for: Modified systemic inflammatory response syndrome and provider gestalt predicting adverse outcomes in children under 5 years presenting to an urban emergency department of a tertiary hospital in Tanzania
Source: Trop Med Health. 2019 Feb 1;47:13. doi: 10.1186/s41182-019-0136-y (PMC6359824; doi:10.1186/s41182-019-0136-y)
Supplement: Supplementary file 1 — Table S1. Baseline Demographics of children evaluating Gestalt. (DOCX 18 kb) [file 41182_2019_136_MOESM1_ESM.docx]

| Table S1: Baseline Demographics of children evaluating Gestalt | | | | | |  |
| --- | --- | --- | --- | --- | --- | --- |
| Variable | **Total**  **N=1350 (%)** | **Healthy**  **N=97 (%)** | **Mild**  **N=546 (%)** | **Moderate**  **N=457 (%)** | **Severe**  **N=250 (%)** | **p-value** |
| Age - months |  |  |  |  |  |  |
| Median | 17 | 24 | 17 | 17 | 12 | 0.0007* |
| Interquartile range | 8 - 32 | 10 – 36 | 8 – 35 | 8 – 31 | 7 – 24 |  |
| Male – no. (%) | 784 (58.1%) | 50 (51.5%) | 333 (61.0%) | 262 (57.3%) | 139 (55.6%) | 0.2274** |
| Female – no. (%) | 566 (41.9%) | 47 (48.  5%) | 213 (39.0%) | 195 (42.7%) | 111 (44.4%) | 0.2274** |
| Findings at presentation |  |  |  |  |  |  |
| Systolic blood pressure – mmHg |  |  |  |  |  |  |
| Median | 102 | 102 | 102 | 102 | 100 | 0.0424* |
| Interquartile range | 92 – 111 | 95 – 115 | 93 – 110 | 93 – 111 | 89 – 109 |  |
| Hypotension – no. (%) | 67 (5.0%) | 0 | 29 (5.3%) | 18 (3.9%) | 20 (8%) | 0.7176** |
| Pulse rate – beats/min |  |  |  |  |  |  |
| Median | 138 | 133 | 133 | 142 | 146 | <0.0001* |
| Interquartile range | 120 – 156 | 116 – 147 | 116 – 150 | 123 – 157 | 127 – 164 |  |
| Tachycardia – no. (%) | 231 (17.1%) | 15 (15.5%) | 68 (12. 5%) | 88 (19.3%) | 60 (24%) | 0.000367** |
| Bradycardia – no. (%) | 26 (1.9%) | 2 (2.1%) | 9 (1.6%) | 7 (1.5%) | 8 (3.2%) | 0.430680** |
| Respiratory rate – breaths/min |  |  |  |  |  |  |
| Median | 32 | 29 | 30 | 32 | 34 | <0.0001* |
| Interquartile range | 26 – 35 | 24 – 32 | 26 – 33 | 28 – 35 | 28 – 42 |  |
| Respiratory distress – no. (%) | 685 (50.7%) | 41 (42.3%) | 255 (46.7%) | 231 (50.5%) | 158 (63.2%) | 0.000069** |
| Axillary temperature – 35.4°C<T>37.9°C-no. (%) | 232 (17.2%) | 5 (5.2%) | 68 (12.5%) | 84 (18.4%) | 75 (30%) | <0.00001** |
| Oxygen saturation - <95% - no. (%) | 98 (7.3%) | 1 (1%) | 18 (3.3%) | 22 (4.8%) | 57 (22.8%) | <0.00001** |
| Responsiveness – Alert – no. (%) | 1274 (94.4%) | 97 (100%) | 543 (99.5%) | 450 (98.5%) | 184 (73.6%) | <0.00001** |
| Voice – no. (%) | 12 (0. 9%) | 0 | 1 (0.2%) | 0 | 11 (4.4%) | <0.0001** |
| Pain – no. (%) | 47 (3.5%) | 0 | 1 (0.2%) | 6 (1.3%) | 40 (16%) | <0.0001** |
| Unresponsive – no. (%) | 17 (1.3%) | 0 | 1 (0.2%) | 1 (0.2%) | 15 (6%) | <0.00001** |

* - p value calculated by Kruskal-Wallis test

** - p value calculated by Chi-Squared test
